# The role of rifampicin within the treatment of *Mycobacterium avium* pulmonary disease

**DOI:** 10.1128/aac.00874-23

**Published:** 2023-10-25

**Authors:** Jodie A. Schildkraut, Jelmer Raaijmakers, Rob Aarnoutse, Wouter Hoefsloot, Heiman F. L. Wertheim, Jakko van Ingen

**Affiliations:** 1 Department of Medical Microbiology, Radboudumc Center for Infectious Diseases, Radboud University Medical Center, Nijmegen, The Netherlands; 2 Department of Pharmacy, Radboudumc Center for Infectious Diseases, Radboud University Medical Center, Nijmegen, The Netherlands; 3 Department of Pulmonary Diseases, Radboudumc Center for Infectious Diseases, Radboud University Medical Center, Nijmegen, The Netherlands; Providence Portland Medical Center, Portland, Oregon, USA

**Keywords:** NTM, nontuberculous mycobacteria, pharmacodynamics, pharmacokinetics, hollow-fibre model

## Abstract

Rifampicin is recommended for the treatment of *Mycobacterium avium* complex pulmonary disease alongside azithromycin and ethambutol. We evaluated the azithromycin-ethambutol backbone with and without rifampicin in an intracellular hollow fiber model and performed RNA sequencing to study the differences in adaptation. In an *in vitro* hollow fiber experiment, we simulated epithelial lining fluid pharmacokinetic profiles of the recommended 3-drug (rifampicin, ethambutol, and azithromycin) or a 2-drug (ethambutol and azithromycin) treatment. THP-1 cells infected with *M. avium* ATCC700898 were exposed to these regimens for 21 days. We determined intra- and extra-cellular bacterial load- and THP-1 cell densities on days 0, 3, 7, 14, and 21, alongside RNA sequencing. The emergence of macrolide resistance was studied by inoculating intra- and extra-cellular fractions of azithromycin-containing Middlebrook 7H10 agar plates. Complete pharmacokinetic profiles were determined at days 0 and 21. Both therapies maintained stasis of both intra- and extra-cellular bacterial populations for 3 days, whilst regrowth coinciding with the emergence of a macrolide-resistant subpopulation was seen after 7 days. THP-1 cell density remained static. Similar transcriptional profiles were observed for both therapies that were minimally influenced by exposure duration. Transcriptional response was slightly larger during 2-drug treatment. Rifampicin did not add to the antimycobacterial effect to the 2-drug therapy or suppression of emergence resistance. RNA transcription was not greatly altered by the addition of rifampicin, which may be due to strong transcriptional influence of azithromycin and host cells. This questions the role of rifampicin in the currently recommended therapy. These findings should be confirmed in clinical trials.

## INTRODUCTION

*Mycobacterium avium* complex (MAC) bacteria are opportunistic pathogens and the most frequent causative agents of nontuberculous mycobacterial pulmonary disease worldwide (1). The recommended treatment of MAC pulmonary disease (MAC-PD) consists of azithromycin, rifampicin, and ethambutol for 12 months after sputum culture conversion, and yields a cure rate of 65% (2, 3). Recurrence rates of MAC-PD are approximately 40%, further underlining the importance of optimizing therapy (4). As evidenced by decreased treatment success rates in macrolide-resistant strains, macrolides are the main driver of treatment efficacy. Rifampicin and ethambutol have little-to-no bactericidal effect but are thought to prevent the emergence of acquired macrolide resistance (2, 5). Rifampicin is a strong CYP3A4 inducer, resulting in lowered exposure to the crucial macrolide antibiotics (6). Given the importance of macrolides in MAC treatment, the pharmacokinetic interaction with rifampicin, rifampicin’s lack of activity against MAC bacteria, and the good outcomes reported for 2-drug ethambutol–macrolide regimens, the benefit of adding rifampicin to MAC treatment has become debatable (7, 8).

We aimed to characterize the effect of adding rifampicin to the ethambutol–azithromycin backbone regimen on MAC-PD treatment outcome, using the hollow fiber infection model supplemented with RNA sequencing.

## MATERIALS AND METHODS

### Bacterial strain, cells, and antibiotics

We purchased the *M. avium* subsp. *homini*s*suis* ATCC 700898 type strain from the American Type Culture Collection (Manassas, VA, USA). Stock vials of the strain were stored at −70°C in trypticase soy broth containing 40% glycerol and were cultured in Middlebrook 7H9 with the addition of 10% OADC 5 days prior to each experiment. THP-1 cells were purchased from the German Collection of Microorganisms and Cell Cultures (DSMZ, Braunschweig, Germany; ACC 16 Lot 32). Stock vials of the cell line were stored at −180°C and thawed for culture in RPMI 1640 with 10% fetal bovine serum (FBS; Thermo Fisher Scientific, Breda, The Netherlands) at 37°C and 5% CO_2_. Azithromycin, rifampicin, and ethambutol were obtained from Sigma-Aldrich (Zwijndrecht, The Netherlands) and dissolved in ethanol, DMSO, and Milli-Q water, respectively. We determined minimum inhibitory concentrations (MICs) of rifampicin, ethambutol, and azithromycin against *M. avium* ATCC 700898 by broth microdilution in cation-adjusted Mueller Hinton broth (CAMHB).

### Hollow fiber system study design and pharmacokinetic parameters

The hollow fiber systems were set up and maintained as described previously (9). Two treatment arms were included, one with rifampicin–ethambutol–azithromycin and one with ethambutol–azithromycin, in addition to an untreated control arm. Pharmacokinetic profiles that are simulated correspond to a human daily dose of 250 mg/day for azithromycin, 15 mg/kg/day for ethambutol, and 600 mg/day for rifampicin. We simulated pharmacokinetic profiles as previously described (9), accounting for a 30% increased azithromycin exposure when not co-administrated with rifampicin (6), and for rifampicin a serum:ELF (Epithelial lung fluid) ratio of 2.6 (10), a 20% serum protein binding and no protein binding in ELF. All arms were included in triplicate. Pharmacokinetic parameters were derived or adapted from pharmacokinetic studies performed in patients with NTM pulmonary disease (Table 1). To simulate the pharmacokinetic profiles, the inflow of fresh medium was set to the half-life of rifampicin. Differences in azithromycin and ethambutol half-life were corrected for by a multiple phase zero order top-up of the drug concentrations in the central reservoir. For a detailed description of the pump settings, see Appendix A.

**TABLE 1:** Pharmacokinetic data simulated in the standard therapy and 2-drug therapy compared with their respective target pharmacokinetic parameters^*a*^

Parameter	Drug	Standard therapy		2-drug regimen	
		Target	Actual	Target	Actual
T_1/2_ (h)	Azithromycin	20 (11)	21.0	20 (12)	24.08
	Ethambutol	10 (13)	10.9	10 (13)	10.84
	Rifampicin	2 (10)	3.6	-	-
T_max_ (h)	Azithromycin	10 (11)	7.3	10 (12)	6.69
	Ethambutol	3 (13)	3.6	3 (13)	3
	Rifampicin	2 (10)	2	-	-
C_ss_ (mg/l)	Azithromycin	3 (11)	3.4 ± 0.3	3.75 (6, 11)	4.3 ± 0.1
	Ethambutol	3 (13)	2.08 ± 0.5	3 (13)	1.9 ± 0.1
	Rifampicin	3.8 (10)	2.06 ± 0.4	-	-
AUC_0–24_ (target)	Azithromycin	-	51.4 (55.58)	-	72.8 (69.48)
	Ethambutol	-	22.9 (38.45)	-	26.6 (38.45)
	Rifampicin	-	14.0 (15.9)	-	-

^*a*^ T_1/2_, simulated half-life; T_max_, time on which C_ss_ is reached; C_ss_, peak concentration in steady-state; AUC_0–24_, area under the concentration-time curve during 24 h and steady state, accompanied by targeting AUC_0–24_ in brackets.

### Pharmacodynamics and emergence of resistance

Bacterial densities (in log_10_ CFU/mL) were determined at prespecified time points (days 0, 3, 7, 14, and 21) by drawing 2 mL samples from each cartridge after thorough mixing. Bacterial densities were determined by inoculating a series of 10-fold diluted samples on Middlebrook 7H10 agar plates (BD Bioscience, Erembodegem, Belgium). To separate intracellular and extracellular bacteria, we spun down the samples (1 mL) at 1,500 rpm for 10 min. The supernatant is used to determine the bacterial density of extracellular bacteria. The pellet is subsequently resuspended in Milli-Q +0.05% tween (1 mL) to lyse THP-1 cells and determine the intracellular bacterial density.

To monitor the emergence of resistance, each sample was inoculated on both drug-free Middlebrook 7H10 agar plates as well as 7H10 agar plates containing azithromycin equal to eight times the initial MIC. In keeping with EUCAST ECOFFS (see www.eucast.org), bacteria able to grow on azithromycin (64 mg/L) containing agar plates were considered resistant.

### Liquid chromatography–mass tandem spectrometry

Concentrations for azithromycin, ethambutol, and rifampicin were determined using a validated triple quadrupole liquid chromatography–mass tandem spectrometry setup (UPLC-MS/MS) (XEVO TQ-S, Waters, Etten-Leur, The Netherlands), as described earlier (9).

### Noncompartmental analysis

Pharmacokinetic data from each system were evaluated using noncompartmental analysis utilizing Phoenix 64 WinNonLin (build 8.1.0.3530) software to determine pharmacokinetic parameters (AUC_0–24_, C_max_, T_max_, and T_1/2_). The linear-up log-down trapezoidal rule was used to determine the area under the concentration-time curve during the dosing interval (24 h, AUC_0–24_). The elimination rate constant was estimated using linear regression of the terminal points of the log-linear concentration-time curve. The parameters C_max_ (maximum concentration) and T_max_ were retrieved by direct inspection of the raw data.

### RNA isolation and sequencing

RNA sequencing of all hollow fiber cartridges was performed at baseline and on days 3, 7, 14, and 21. RNA isolation was performed at each time-point using the NucleoSpin RNA kit (Machery Nagel, Düren, Germany) with slight modification. In short, 1 mL from each cartridge was collected, cells and bacteria were pelleted and resuspended in 350 µL lysis buffer, thereby lysing host cells. Subsequently, each sample was centrifuged for 5 min at maximum speed and 175 µL supernatant containing unwanted host RNA was removed. The remaining pellet and supernatant were transferred to a microcentrifuge tube containing one scoop of glass beads and an additional 175 µL of lysis buffer was added. Samples were then subjected into two rounds of cold shock followed by bead-beating at 7,000 rpm for 30 s. RNA was then isolated following the manufacturer’s protocol. RNA integrity and concentration were then measured on a TapeStation 2200 (Agilent, Santa Clara, USA) and Qubit to ensure sufficient integrity and concentration (>10 ng). RNA library preparation was performed using the Illumina Stranded Total RNA Prep with Ribo-Zero Plus kit following the manufacturer’s instructions. Finished libraries were measured on a TapeStation 2200 to ensure the correct length. Finally, enrichment of bacterial reads was then performed using a custom myBaits capture kit (Arbor Biosciences, Ann Arbor, USA) following the manufacturer’s protocol (see supplementary file for design). Libraries were then run in paired-end 2 × 75 bp mode on a NextSeq 500 (Illumina, Sand Diego, USA).

### Differential gene expression analysis

All reads were mapped to the well-annotated *M. avium* subsp. *hominissuis* 109 genome using STAR (14). Differential expression analysis was performed using the DESeq2 R package and cut-offs were defined as Log2 fold change ≥ 2 or ≤ −2, *P*-value ≤  0.05, correction for multiple guessing was performed by the Benjamini and Hochberg principle (15, 16). Subsequently all gene ontology (GO) terms were retrieved from the publication by Matern et al. and an enrichment analysis was performed using the topGO R package (17). Enriched GO-terms were identified using a Fishers exact test weighted for GO hierarchy with a *P*-value ≤ 0.05. Enriched GO terms containing only one DEG were disregarded.

## RESULTS

### MICs, pharmacodynamics, and emergence of resistance

**Fig 1:**
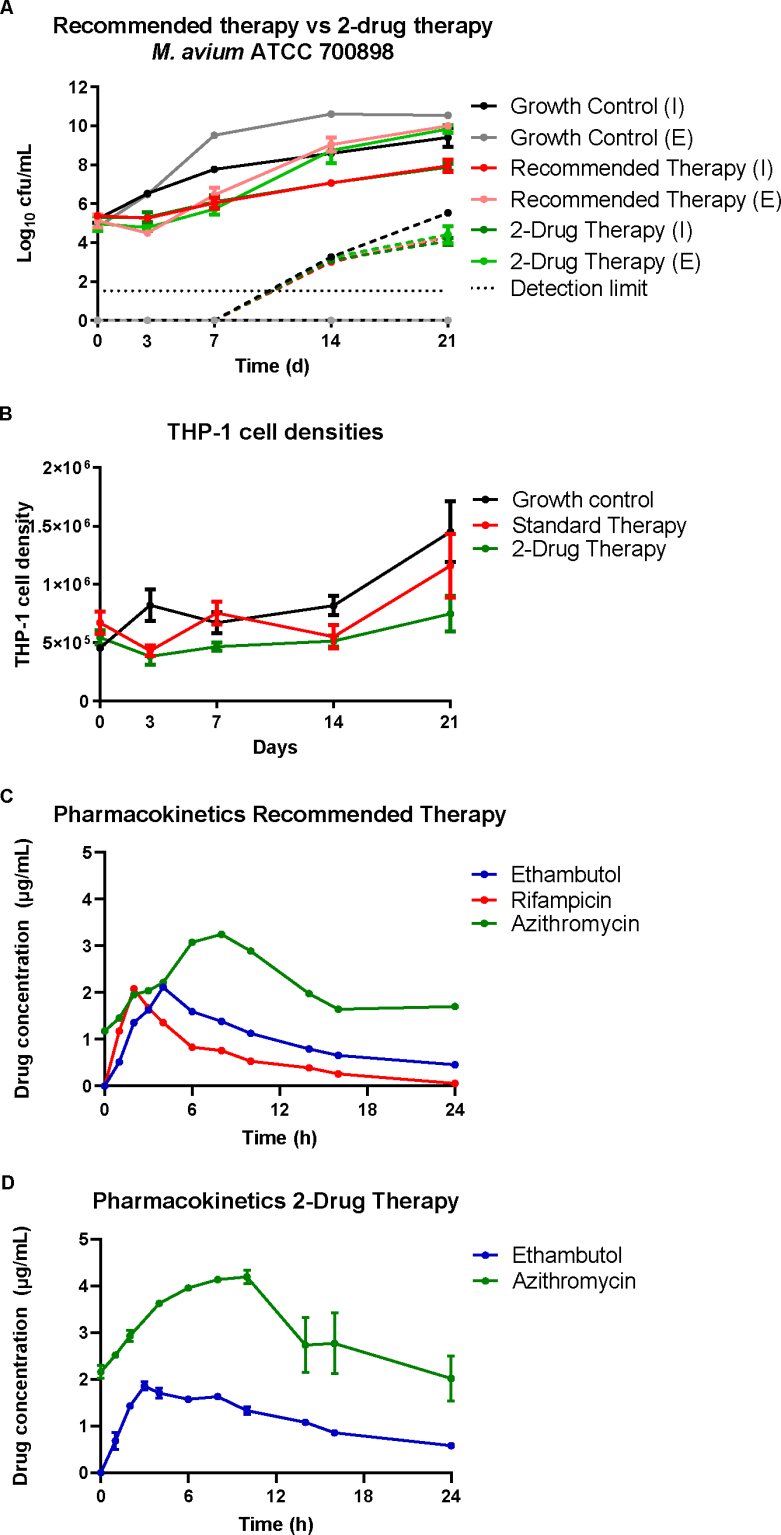
(A) Bacterial densities over time (solid lines) and emergence of macrolide resistance (dotted lines). (**B**) THP-1 cell densities over time. (**C**) Pharmacokinetic profiles at steady state of the standard therapy. (**D**) Pharmacokinetic profiles at steady state of the 2-drug therapy. Dosing regimen: azithromycin 250 mg/day; ethambutol 15 mg/kg/day; rifampicin 600 mg/day.

MICs for rifampicin, ethambutol, and azithromycin, determined by broth microdilution in CAMHB were 4, 4, and 8 mg/L, respectively, and did not change over the course of treatment. In the hollow fiber infection model, both regimens were able to suppress bacterial growth for up to 3 days, after which neither proved able to sustain its efficacy (Fig. 1A). We observed extracellular exponential regrowth in all experimental arms from day 7 onwards. This growth coincided with the emergence of azithromycin resistance as determined by bacterial counts grown on azithromycin-containing plates for both regimens. Eventually, a plateau phase was reached. A linear growth rate was seen for intracellular bacteria in all arms. On day 14, one growth control was contaminated and was excluded from further analysis.

### Pharmacokinetics

The mimicked azithromycin pharmacokinetic profiles were similar to the targeted pharmacokinetic profiles ((Fig. 1C and D; Table 1). A slightly higher azithromycin exposure ratio between the two arms was mimicked (1.40) than anticipated (1.30). A lower ethambutol C_max_ was reached than targeted in all experimental arms. We simulated a lower rifampicin C_max_ and a longer half-life than anticipated. Total exposures met the target exposures.

### Differentially expressed genes

The number of mycobacterial reads recovered after enrichment was lowest at baseline and increased over time as the proportion of bacteria relative to THP-1 cells increased (Table S1).

**Fig 2:**
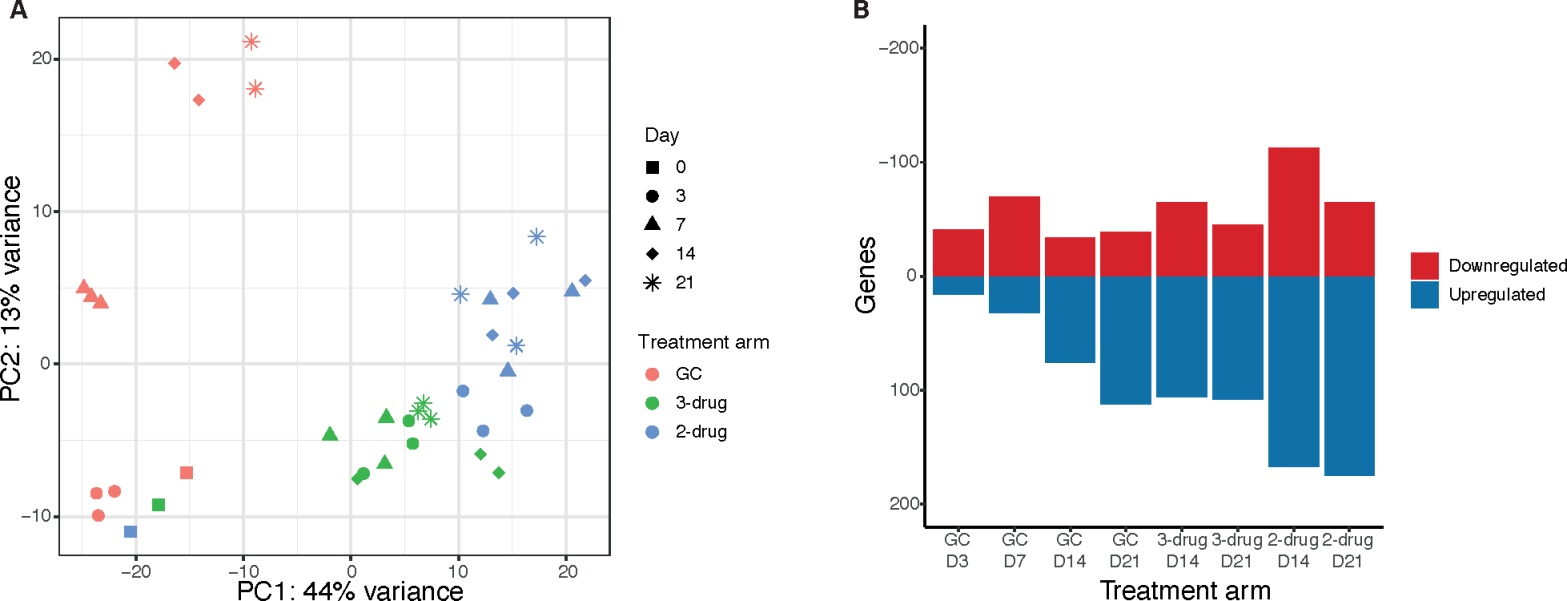
(A) PCA plot of the transcriptomic profile of all included time-points per treatment arm. (**B**) Bar chart illustrating the number of up- and down-regulated genes at each time point for the growth control and on days 14 and 21 for the 2-drug (azithromycin–ethambutol) and 3-drug (azithromycin–ethambutol–rifampicin) arm.

We observed three important changes in the transcriptional profiles. First, principal component analysis showed that the antibiotic treatment led to the greatest amount of variance (*x*-axis, 44%), while gradual changes in the growth control arm was the second largest driver of variance (*y*-axis, 13%; Fig. 2A). Second, the effect of the 2-drug regimen on gene transcription was larger than that of the 3-drug regimen. Third, exposure duration and growth phase only minimally effect transcription, as indicated by the gradual differentiation over time corresponding with bacterial growth phase in the control arm, with each time point moving further along the *y*-axis and, in contrast, the similarity between days 3 and 21 in the 2- and 3-drug treatment arms.

We then determined the number of DEGs relative to baseline for each time point in the untreated arm (Fig. 2B). In addition, we determined the number of DEGs between the 3-drug and the 2-drug arms for days 14 and 21 when read counts were sufficient (Table S1; Fig. 2B). The 2-drug regimen showed slightly higher numbers of DEGs when compared to the control arm than the 3-drug regimen on days 14 and 21. Finally, the number of genes upregulated in the 2-drug arm when compared to the 3-drug arm was slightly larger on day 14 than on day 21.

### GO enrichment of differentially expressed genes

We then performed GO enrichment of DEGs between the control, 2- and 3-drug arms for days 14 and 21, after log-phase growth. On day 14 in the 2-drug arm an enrichment of genes involved in glycerol-3-phosphate metabolism, glycolipid biosynthesis, and fatty acid synthase activity was seen. In addition, enrichment was seen of ATP-binding cassette (ABC) transporter genes and genes related to the NADH dehydrogenase. On day 21, only an enrichment of three non-specific GO terms, immune response, integral component of membrane and extracellular region were seen. In the 3-drug arm, an enrichment of genes involved in protein autophosphorylation, menaquinone biosynthesis, acetyl-CoA carboxylase activity, and thiamine pyrophosphate binding was seen. Again, at day 21 in the 3-drug arm enrichment of only three GO terms, transcription antitermination, integral component of membrane, and FAD bindings was seen.

In addition, we performed a GO analysis of all control arm time points to study the development of the control population over time (Table S2). GO enrichment of different timepoints within the control arm showed enrichment of genes involved in different metabolic pathways at different time points during growth. On day 3, when the bacterial population was increasing rapidly, an increase in genes related to DNA-templated transcription termination and carboxylic acid metabolic processes were seen. On day 7, when growth began to slow, these pathways were no longer enriched but an enrichment of genes involved in “*de novo*” UMP biosynthesis and methylisocitrate lyase activity was seen. On day 14, as the plateau phase of growth was reached, an enrichment of genes involved in pantothenate biosynthesis and acetyl-CoA carboxylase activity, unfolded protein binding, and heme binding was seen. Finally, on day 21 an enrichment of genes involved in pantothenate biosynthesis and acetyl-CoA carboxylase activity was still seen. However, this was also accompanied by an enrichment of genes involved in lipid metabolic processes, thiamine metabolism, leucine biosynthesis, and 3-isopropylmalate dehydratase activity.

Finally, GO enrichment of DEGs between the 2- and 3-drug arms resulted in three enriched GO terms (phosphorelay signal transduction system, signal transduction by protein phosphorylation, and intracellular) on day 14, while none were identified on day 21.

## DISCUSSION

Using an *in vitro* hollow fiber model that simulates epithelial lining fluid pharmacokinetics, we observed that rifampicin adds no killing capacity to the ethambutol–azithromycin backbone in the treatment of MAC-PD. Both therapies proved equally effective and able to suppress bacterial growth for up to 3 days, after which the bacteria steadily grew with the emergence of azithromycin-resistance. No large differences in transcription were seen between both therapies.

We did not see an increased bacterial killing despite the 40% higher azithromycin exposure as a result of the absence of rifampicin-induced CYP3A4-mediated pharmacokinetic interactions (6). A retrospective case series from South Korea has suggested that higher azithromycin exposures are associated with favorable microbiological treatment outcomes (18). On the other hand, previous hollow fiber model experiments have suggested that the currently recommended doses of azithromycin are too low to exert a significant antimycobacterial effect (19); the increase in exposure in our study may still be too modest to yield an increased effect. Also, rifampicin did not impact the emergence of macrolide resistance in the current experiment. The recommendation to use rifampicin on top of ethambutol to prevent macrolide resistance (2) stems from a single retrospective case series in MAC-PD, which cited two studies in HIV patients that assessed this role of rifamycins but of which only one showed a marginal positive effect on preventing macrolide-resistance in a subgroup of patients (20 – 22). As rifampicin lowers macrolide and other drug exposures, causes significant toxicity, and is of unproven efficacy in MAC-PD regimens, removing it from the regimen might be beneficial in select categories of MAC-PD patients (23). Recent retrospective case series have confirmed that a 2-drug therapy has similar outcomes than to the recommended therapy for mild or moderate nodular-bronchiectatic MAC-PD, comparable to our *in vitro* observation (7, 8).

Theoretically, rifampicin’s poor efficacy might be overcome by attaining higher exposures. In a recent time-kill kinetics study, a combination of clarithromycin–ethambutol–rifampicin was more effective than a clarithromycin–ethambutol regimen, showing an effect size of 89.1 and 64.6 CFU/mL·day^−1^, respectively (24). However, static concentrations of two times the MIC were used, far exceeding clinically observed concentrations. For rifampicin, that results in concentrations 8-fold higher than the C_max_ simulated in the current study. To reach that C_max_ in the lungs, considering the serum:ELF ratio of 2.6, a 50 mg/kg dose of rifampicin must be administered, which proved intolerable in a recent tuberculosis trial (25).

A 2-drug ethambutol–azithromycin therapy also proved unable to suppress bacterial growth for more than 7 of the 28 days in a previous study employing an intracellular hollow fiber model, but mimicking serum pharmacokinetics (26). Yet, the investigators link the microbiological failure to the emergence of ethambutol resistance, as at the final day of the experiment (day 28), they noticed a 100% ethambutol-resistant subpopulation and no emergence of macrolide resistance. This latter finding contrasts with the emergence of azithromycin resistance we observed from day 7 onwards; the absence of macrolide resistance in the prior study may result from using plates with higher concentrations (96 mg/L vs 64 mg/L in our study) of azithromycin. We did not assess ethambutol resistance.

### RNA sequencing indicates a 2-drug regimen does not lead to more resistance

The transcriptomic profile of *M. avium* exposed to either the 2- or 3-drug regimen is highly similar, although the 2-drug regimen shows a slightly larger response accompanied by a higher number of DEGs (Fig. 2A and B). In addition, in contrast to the growth control arm, the transcriptional profile of the treated arms only differed minimally over time. The stability of the transcriptomic profile in the presence of antibiotics over time suggests two things. First, the duration of exposure does not greatly influence transcription as minimal numbers of DEGs and enriched GO terms are found between time points. Secondly, the changes in transcription seen in the growth control over time are not essential to outgrowth within the HFM as they are not found in treatment arms. Furthermore, the similarity between the transcriptomic responses in both arms can likely be explained by the presence of azithromycin and THP-1 cells within the cartridge, while the larger size of the response in the 2-drug arm likely results from the higher concentration of azithromycin. Previous studies have shown that of all antibiotics, the macrolide antibiotic clarithromycin has the largest effect on transcription, while the effect of both rifampicin and ethambutol is much smaller (27, 28). Because azithromycin and clarithromycin have the same mechanisms of action the response to azithromycin alone is likely comparable to that of clarithromycin. These differences between the arms indicate an exposure-dependent effect of macrolide antibiotics on transcription, which is substantiated by the fact that downregulation of the NADH dehydrogenase, previously shown to be a key transcriptional response to clarithromycin in both *M. avium* and *M. abscessus* (28, 29), is only seen in the 2-drug arms. Furthermore, the THP-1 cells in the hollow fiber cartridge likely also influence the response of *M. avium* to the antibiotic exposure; a previous study showed using *M. avium* and human monocyte-derived macrophages showed that in the intracellular environment, the effect of macrolide antibiotics is much less pronounced, and the host-pathogen interaction dominates the transcriptomic response (27). Together, the presence of THP-1 cells and azithromycin likely drive the transcriptomic response of both the 2- and 3-drug arms, and thus the omission of rifampicin in treatment will likely not lead to greater adaptive flexibility, and thereby resistance within *M. avium*.

This study has several important limitations. The pharmacokinetic profiles are based on ELF and are therefore likely to represent the nodular/bronchiectatic diseases rather than the fibrocavitary MAC-PD as cavity wall and core concentrations of relevant drugs are lower (30). In our experiment, a 50% lower C_max_ and a prolonged half-life of rifampicin were simulated than anticipated, possibly by clogging of the inlet filters. Total exposure as measured by the AUC_0–24_ was similar between the target (14.7 mg/L·h^−1^) and actually simulated (14.0 mg/L·h^−1^). The AUC/MIC ratio of rifampicin has previously been shown to be the pharmacodynamic parameter that best correlated with bacterial count reduction in mice (31), and clinical studies have also used AUC/MIC as a predictor for efficacy (6, 32). Therefore, we evaluate that our exposure is sufficient for the interpretation of our results.

Furthermore, due to the limited sample volume within the hollow fiber cartridges, the RNA yield of the bacterial population is limited, particularly during the first week when the bacterial population is small, this led to limited sequencing depth at many time points. This is particularly so in the first week of treatment when the bacterial population is small. Due to the limited depth of sequencing, we believe that small changes in transcription likely went unmeasured, and, therefore, in this study we focused on the differences on a transcriptome-wide scale and not on in-depth pathways. Additionally, the limited genomic annotation of *M. avium* means that many transcriptional changes cannot be interpreted functionally. Finally, further clinical validation of these *in vitro* findings is still required, although a comparative clinical trial studying the additional effect of rifampicin is currently ongoing (Clinicaltrial.gov; registration number: NCT03672630).

In conclusion, a 2-drug regimen of ethambutol and azithromycin is as efficacious as the recommended 3-drug regimen of rifampicin, ethambutol, and azithromycin in a hollow fiber model simulating nodular-bronchiectatic MAC-PD treatment. This is in line with recent clinical studies describing the efficacy of the 2-drug regimen for MAC-PD (7, 33). This questions rifampicin’s role in the recommended MAC-PD regimen, particularly in milder manifestations, although these findings remain to be confirmed in ongoing clinical trials. Furthermore, the absence of large differences in transcription between the 2- and 3-drug regimen and the equal time to emergence and population size of azithromycin-tolerant bacteria indicate that 2-drug regimens will likely not lead to increased levels of resistance due to more permissibility in adaption to the antibiotic stress.

## SUPPLEMENTAL MATERIAL

Supplemental material 2 vs 3 drug aac.00874-23-s0001.docx Contains supplemental tables regarding RNA seq read, hollow-fibre pump settings and drug concentration method (LC-Ms/Ms).

ASM does not own the copyrights to Supplemental Material that may be linked to, or accessed through, an article. The authors have granted ASM a non-exclusive, world-wide license to publish the Supplemental Material files. Please contact the corresponding author directly for reuse.
