# Supplementary material for: The role of rifampicin within the treatment of Mycobacterium avium pulmonary disease
Source: Antimicrob Agents Chemother. 2023 Oct 25;67(11):e00874-23. doi: 10.1128/aac.00874-23 (PMC10649009; doi:10.1128/aac.00874-23)
Supplement: Supplemental material 2 vs 3 drug — Contains supplemental tables regarding RNA seq read, hollow-fibre pump settings and drug concentration method (LC-Ms/Ms). [file aac.00874-23-s0001.docx]

**Supplemental material**

**Supplementary table 1. Number of reads per time point in each arm.**

| **D0** | | **D3** | | **D7** | | **D14** | | **D21** | |
| --- | --- | --- | --- | --- | --- | --- | --- | --- | --- |
| **Sample** | **Reads** | **Sample** | **Reads** | **Sample** | **Reads** | **Sample** | **Reads** | **Sample** | **Reads** |
| C | 45377 | C1 | 419998 | C1 | 574077 | C1 | 1789375 | C1 | 6616546 |
| R | 211460 | C2 | 425441 | C2 | 452512 | C3 | 7312601 | C3 | 4130096 |
| S | 72585 | C3 | 754911 | C3 | 1147305 | R1 | 2786829 | R1 | 3086761 |
|  |  | R1 | 176426 | R1 | 784766 | R2 | 1066021 | R2 | 5977601 |
|  |  | R2 | 169216 | R2 | 298005 | R3 | 2654507 | R3 | 578559 |
|  |  | R3 | 250164 | R3 | 236398 | S1 | 782783 | S1 | 882820 |
|  |  | S1 | 67945 | S1 | 39623 | S2 | 514326 | S2 | 1816807 |
|  |  | S2 | 41682 | S2 | 61538 | S3 | 598749 | S3 | 1715400 |
|  |  | S3 | 43643 | S3 | 252620 |  |  |  |  |

**Supplementary table 2. Enriched GO terms per condition and time point.**

| Sample | Ontology | GO Term | Annotated to GO term | Significant |
| --- | --- | --- | --- | --- |
| GC-D3 | BP | DNA-templated transcription, termination | 14 | 2 |
| GC-D3 | BP | carboxylic acid metabolic process | 309 | 6 |
| GC-D3 | MF | 3-hydroxyacyl-CoA dehydrogenase activity | 4 | 2 |
| GC-D3 | MF | monooxygenase activity | 118 | 6 |
| GC-D3 | MF | iron ion binding | 93 | 4 |
| GC-D3 | MF | FAD binding | 24 | 2 |
| GC-D7 | BP | 'de novo' UMP biosynthetic process | 7 | 2 |
| GC-D7 | MF | methylisocitrate lyase activity | 4 | 2 |
| GC-D14 | BP | pantothenate biosynthetic process | 6 | 2 |
| GC-D14 | BP | regulation of cell shape | 24 | 3 |
| GC-D14 | CC | small ribosomal subunit | 8 | 2 |
| GC-D14 | MF | acetyl-CoA carboxylase activity | 3 | 2 |
| GC-D14 | MF | unfolded protein binding | 12 | 3 |
| GC-D14 | MF | heme binding | 82 | 6 |
| GC-D21 | BP | protein folding | 15 | 5 |
| GC-D21 | BP | protein quality control for misfolded or incompletely synthesized proteins | 3 | 2 |
| GC-D21 | BP | lipid metabolic process | 187 | 7 |
| GC-D21 | BP | pantothenate biosynthetic process | 6 | 2 |
| GC-D21 | BP | thiamine metabolic process | 7 | 2 |
| GC-D21 | BP | thiamine-containing compound biosynthetic process | 7 | 2 |
| GC-D21 | BP | leucine biosynthetic process | 8 | 2 |
| GC-D21 | CC | small ribosomal subunit | 8 | 2 |
| GC-D21 | MF | unfolded protein binding | 12 | 5 |
| GC-D21 | MF | 3-isopropylmalate dehydratase activity | 2 | 2 |
| GC-D21 | MF | ATP binding | 376 | 23 |
| GC-D21 | MF | acetyl-CoA carboxylase activity | 3 | 2 |
| GC-D21 | MF | heat shock protein binding | 3 | 2 |
| R-14C | BP | glycerol-3-phosphate metabolic process | 5 | 2 |
| R-14C | BP | biosynthetic process | 1074 | 22 |
| R-14C | BP | metabolic process | 2706 | 74 |
| R-14C | BP | ATP synthesis coupled electron transport | 8 | 2 |
| R-14C | BP | glycolipid biosynthetic process | 22 | 3 |
| R-14C | BP | immune response | 21 | 2 |
| R-14C | CC | integral component of membrane | 1081 | 61 |
| R-14C | CC | ATP-binding cassette (ABC) transporter complex | 35 | 4 |
| R-14C | MF | erythronolide synthase activity | 3 | 2 |
| R-14C | MF | phosphopantetheine binding | 25 | 4 |
| R-14C | MF | NADH dehydrogenase (ubiquinone) activity | 14 | 3 |
| R-14C | MF | 3-oxoacyl-[acyl-carrier-protein] synthase activity | 8 | 2 |
| R-14C | MF | fatty acid synthase activity | 70 | 7 |
| R-21C | BP | immune response | 21 | 2 |
| R-21C | CC | integral component of membrane | 1081 | 22 |
| R-21C | CC | extracellular region | 187 | 6 |
| S-14C | BP | protein autophosphorylation | 6 | 3 |
| S-14C | BP | signal transduction by protein phosphorylation | 21 | 3 |
| S-14C | BP | pathogenesis | 78 | 6 |
| S-14C | BP | cell adhesion | 9 | 2 |
| S-14C | BP | menaquinone biosynthetic process | 9 | 2 |
| S-14C | BP | cellular response to chemical stimulus | 26 | 3 |
| S-14C | CC | integral component of membrane | 1081 | 45 |
| S-14C | MF | acetyl-CoA carboxylase activity | 3 | 2 |
| S-14C | MF | thiamine pyrophosphate binding | 16 | 3 |
| S-21C | BP | transcription antitermination | 11 | 2 |
| S-21C | CC | integral component of membrane | 1081 | 24 |
| S-21C | MF | FAD binding | 24 | 3 |
| S-14-R | BP | phosphorelay signal transduction system | 48 | 3 |
| S-14-R | BP | signal transduction by protein phosphorylation | 21 | 2 |
| S-14-R | CC | intracellular | 704 | 9 |

***Supplementary table 3. Syringe pump settings for daily infusions***

| Standard Regimen | | | |
| --- | --- | --- | --- |
| Drug | Infusion Volume (mL/h) | Time (hh:mm) | Syringe concentration (mg/L) |
| Azithromycin | 1.4 | 10:05 | 350 |
|  | 0.72 | 09:55 |  |
|  | 0.15 | 04:00 |  |
| Ethambutol | 1.2 | 03:05 | 500 |
|  | 0.5 | 04:55 |  |
|  | 0.35 | 12:00 |  |
|  | 0.15 | 04:00 |  |
| Rifampicin | 1.3 | 02:20 | 400 |
|  | Delay | 21:40 |  |
| 2-drug regimen | | | |
| Azithromycin | 1.4 | 10:05 | 550 |
|  | 0.72 | 09:55 |  |
|  | 0.15 | 04:00 |  |
| Ethambutol | 1.2 | 03:05 | 500 |
|  | 0.5 | 04:55 |  |
|  | 0.35 | 12:00 |  |
|  | 0.15 | 04:00 |  |
| System parameters | | | |
| Pump inflow | 1.9 mL/h | Distribution Volume | 322 mL |

**Supplementary Methods**

**Bacterial enumeration**

Before sampling, the cartridges were vigorously mixed using two 20 ml syringes, and 2 ml suspension was removed from the cartridge. This sample (1 ml) was spun down for 10 min at 1500rpm, and supernatant was removed for extracellular bacterial enumeration

**Hollow fiber system**

Diluent medium also consisting of RPMI640 with 2% FBS was pre-filtered using autoclaved Culture Guard 0.22 µm filters (Repligen, Waltham, MA, USA) and pumped into the system using peristaltic pumps (530S with 313x pump head extensions, Watson Marlow, Barendrecht, the Netherlands) with a speed of 1.9mL/min. Tubing to and from the system was Pumpsil 1.6mm Bore x 1.6mm wall thickness platinum cured silicone tubing (Watson Marlow, Barendrecht, NL) fitted with 3/32 ID luer fittings.

**Pharmacokinetic Measurements**

Time points were chosen to capture peak drug concentrations as well as trough levels, in order to verify that pharmacokinetic parameters were correctly simulated. We first flushed the filtered clave connector by removing 2 ml medium through it, and then taking a 1 ml sample, ensuring only fresh medium was sampled. Samples were processed immediately.

**Calculations**

Pump rates in both drug syringes and media pumps were calculated using standard pharmacokinetic equations. To mimic the half-lives of the different drugs, the pumps were set to eliminate the drug with the shortest half-life accurately. To achieve distinct other half-lives, we then calculated how much additional drug would need to be injected per time unit of the other drugs to artificially prolong their apparent half-lives. The elimination rate constant k was calculated with k_e_ = ln(2)/t_1/2_, where t_1/2_ is the half-life of the drug with the shortest *in vivo* half-life. The clearance CL per hour was calculated using CL = k_e_ * V_D_, where V_D_ was the total volume of the system. During infusion, we calculated the drug concentration C using C = k_0_/CL * (1- e^-ke∙t^), where k_0_ is the infusion rate and k is the elimination rate constant calculated before. The concentration C of drug at time T after infusion was calculated using C_t_ = C_0_ * e^-ke∙t^ where C_0_ is the concentration at T_max_ and k_e_ is the elimination rate constant. We assumed that after 5 x half-lives drug concentrations are undetectably low. The used calculation sheet can be assessed as Supplementary datasheet 1. To account for natural fluctuations in inflow, outflow and drug distribution, we added 10% on top of the calculated syringe concentration and rounded to a convenient concentration.

**Pharmacokinetic Measurement protocols**

Azithromycin Pharmacokinetic Sample measurement protocol

**Reference materials**

- Azithromycin European Pharmacopoeia (EP) Reference Standard (Sigma-Aldrich, Zwijndrecht, The Netherlands)
- Azithromycin [^13^C, ^2^H_3_] (Alsachim, Illkirch-Graffenstaden, France)

**Chemicals and solvents**

- Acetonitrile, Hypergrade for LC-MS (Merck, Darmstadt, Germany)
- Methanol, Absolute ULC/MS (Biosolve, Valkenswaard, The Netherlands)
- Dimethyl sulfoxide (Merck, Darmstadt, Germany)
- Formic acid, 98-100% for LC-MS (Merck, Darmstadt, Germany)
- RPMI Medium 1640 (Thermo Fisher Scientific, Breda, The Netherlands)
- Fetal bovine serum (Thermo Fisher Scientific, Breda, NL)

**Stocks and work solutions**

Three independent stock solutions (for calibrators, quality controls, and a reserve), were prepared at a concentration of 1mg/ml and stored at -40 °C for Azithromycin (AZT). AZT stocks were dissolved with methanol.

The labeled internal standard stock is prepared and stored in the same manner as the unlabeled compound stocks, at 1 mg/ml.

From the labeled internal stock solutions, a protein precipitation (PP) solution is made with a concentration of 0.1 mg/L in acetonitrile and stored at -40°C.

**Calibration and quality control solutions**

For the preparation of the calibration curve, one of the stock solutions was used and diluted with acetonitrile to achieve seven calibration solutions 0.30–0.54–1.95–3.75–7.5–15-30 mg/L AZT and 1.0-4.0-7.5-15-30-60 mg/L MINO.

Quality control (QC) solutions are made at three levels (low, medium and high) from a different stock than the calibration solutions at 0.4–6-20 mg/L for AZT and 2.3 -15-50 mg/L MINO. During sample preparations these working solutions are further diluted (factor 20) in RPMI 1640 + 2%. All solutions were stored at −80 °C until analysis and are stable for at least 7 months.

**Equipment and settings**

**Supplementary table 4. Pump settings used for ACQUITY UPLC H-Class QSM for azithromycin pharmacokinetic determinations**

| **Pump settings (ACQUITY UPLC H-Class QSM; Waters, Milford, MA, USA)** | | | |
| --- | --- | --- | --- |
| **Time (min)** | **Flowrate (ml/min)** | **% A**  Water + 0.1% formic acid | **% B**  Acetonitrile + 0.1% formic acid |
| 0 | 0,3 | 98 | 2 |
| 1.00 | 0,3 | 30 | 70 |
| 2.00 | 0.6 | 30 | 70 |
| 3.00 | 0,1 | 30 | 2 |
| 3.25 | 0,3 | 98 | 2 |
| 5.50 | 0,3 | 98 | 2 |
| 6.00 | 0.3 | 98 | 2 |
|  | | | |
| Seal wash: 90/10 water/acetonitrile | | | |
| Seal wash period: 5 min | | | |

**Supplementary table 5. Autosampler and column oven settings for azithromycin pharmacokinetic determinations**

| **Autosampler and column oven (ACQUITY UPLC H-Class SM-FTN; Waters, Milford, MA, USA)** |
| --- |
| Purge solvent: 95/5 water/acetonitrile + 0.1% formic acid |
| Wash solvent: 40/60 water/acetonitrile + 1% formic acid |
| Injection volume: 1 µl |
| Pre-inject wash time: 5 sec |
| Post-inject wash time: 5 sec |
| Needle placement: 4mm |
| Column Temperature 25 ± 1°C |
| Column: Xbridge C18 3.5um 2,1x50mm |
| Temp. tray: 10°C |

**Supplementary table 6. Ionspray source used for azithromycin pharmacokinetic determinations**

| **Ionspray source** |
| --- |
| Capillary: 2 kV |
| Polarity positive ion mode |
| Desolvation temperature: 500 °C |
| Desolvation gas flow: 950 L/hr |
| Cone flow: 10 L/hr |

**Supplementary table 7. Mass spectrometer settings used for azithromycin and pharmacokinetic determinations**

| **MS settings (XEVO TQS-micro; Waters, Milford, MA, USA)** | | | | | |
| --- | --- | --- | --- | --- | --- |
| **Compound Name** | **Parent (m/z)** | **Daughter (m/z)** | **Dwell (s)** | **Cone (V)** | **Collision (V)** |
| Azithromycin | 749.5 | 116.0 | 0.1 | 16 | 48 |
| Azithromycin [^13^C, ^2^H_3_] | 753.4 | 158.1 | 0.1 | 46 | 36 |
|  | | | | | |
| **Ionspray source** | | | | | |
| Capillary: 2 kV | | | | | |
| Polarity: positive ion mode | | | | | |
| Desolvation temperature: 500 °C | | | | | |
| Desolvation gas flow: 950 L/hr | | | | | |
| Cone flow: 10 L/hr | | | | | |

**Accessories and disposables**

***Supplementary table 8. Aaccessories and disposable used for rifampicin and ethambutol pharmacokinetic determinations***

| **Name** |
| --- |
| Finnpipette® Air displacement pipette 50-200 µl (ThermoFisher Scientific, Breda, The Netherlands) |
| Finnpipette® Air displacement pipette 5 - 40 µl (ThermoFisher Scientific, Breda, The Netherlands) |
| Finnpipette® Pipette tips 250 µl (ThermoFisher Scientific, Breda, The Netherlands) |
| HandyStep ® Repeater pipette (Brand, Wertheim, Germany) |
| HandyStep ® Repeater pipette tip 5 ml (Brand, Wertheim, Germany) |
| Autosampler vials: TPX micro-vial ND9 insert int. 0.2 ml TPX clair 32x11.6mm (VWR, Amsterdam, The Netherlands) |
| Autosampler caps: PP Screwcap 9mm pre-slit septum (VWR, Amsterdam, The Netherlands) |
| Safe-Lock tube 1.5 ml (Eppendorf, Nijmegen, The Netherlands) |
| DVX-2500 Multi-Tube Vortexer (VWR, Amsterdam, The Netherlands) |

**Sample preparation**

1. Thaw and mix the (pre) created calibrators and qc’s
2. In safe-lock tubes, dilute the calibrators and qc’s a factor 20 in RPMI 1640 + 2% FBS (e.g. 30 µl in 570 µl RPMI 1640 + 2% FBS)
3. Mix the diluted calibrators and qc’s with a multi-tube vortex
4. Pipette in a safe-lock tube: 50 µl sample, diluted QC or calibrator and add 150 µl PP solution
5. Mix with a multi-tube vortex 20 sec, speed 2500 rpm
6. Centrifuge 5 min.(18.620 g)
7. Close the autosampler vial with a pre-slit cap
8. Inject the sample(s) on the chromatographic separation system

**Calculations**

Acquired data was processed using Waters TargetLynx software (version 4.1). The calibration curves were linear fitted as ratio signal response compound peak area and internal standard peak area versus concentration, also a 1/x^2^ weighing factor was used.

**Chromatogram**


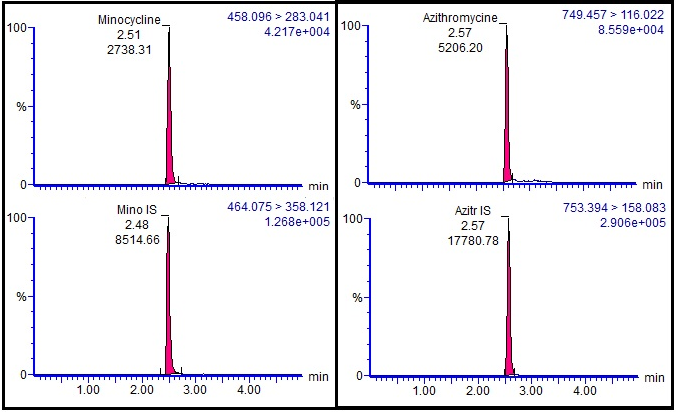


**Supplementary figure 1. Chromatogram of the lowest calibrator with internal standard**

**Validation results**

***Supplementary table 9. Within – and between run accuracy and precision for ethambutol (EMB) and rifampicin (RIF).***

|  | Conc. | Within run (n=5) | | Between run (n=15) | |
| --- | --- | --- | --- | --- | --- |
|  | (mg/l) | Accuracy (%) | Precision (%) | Accuracy (%) | Precision (%) |
| AZIT | 0.0150  0.0190  0.301  1.004  1.507 | 97.9  91.8  94.5  93.4  95.9 | 8.69  4.42  5.43  7.61  8.12 | 99.1  94.3  98.1  97.6  98.5 | 8.69  3.88  3.36  2.85  8.12 |

**Rifampicin & Ethambutol pharmacokinetic Sample measurement protocol**

**Reference materials**

- Ethambutol dihydrochloride salt, antimycobacterial (Sigma-Aldrich, Zwijndrecht, The Netherlands)
- Ethambutol ^2^H_4_ (Alsachim, Illkirch-Graffenstaden, France)
- Rifampicine >97% powder (Sigma-Aldrich, Zwijndrecht, The Netherlands)
- Rifampicine ^2^H_8_ (Alsachim, Illkirch-Graffenstaden, France)

**Chemicals and solvents**

- Acetonitrile, Hypergrade for LC-MS (Merck, Darmstadt, Germany)
- Methanol, Absolute ULC/MS (Biosolve, Valkenswaard, The Netherlands)
- Ascorbic acid, Ph.Eur. (Spruyt-Hillen, IJselstein, The Netherlands)
- Formic acid, 98-100% for LC-MS (Merck, Darmstadt, Germany)
- Ammonium formate, Eluent additive for LC-MS (Sigma Aldrich, Zwijndrecht, The Netherlands)
- RPMI Medium 1640 (Thermo Fisher Scientific, Breda, The Netherlands)
- Fetal bovine serum (Thermo Fisher Scientific, Breda, NL)

**Stock and work solutions**

Three independent stock solutions (for calibrators, quality controls, and a reserve), were prepared and stored at -40 °C. Ethambutol (EMB) stock 1mg/ml was prepared in water and rifampicin (RIF) stock 2 mg/ml in methanol/water 80/20 v/v with 0.16mg/ml ascorbic acid.

The labeled internal standard stocks are made by dissolving the compounds in the same manner as the unlabeled compounds stocks, only at 1 mg/ml.

From the labeled internal stock solutions, a protein precipitation (PP) solution is made with 0.1 mg/L EMB and 0.1 mg/L RIF in acetonitrile/methanol 75/25 v/v. All stocks and solutions were stored at -40°C.

**Calibration and quality control solutions**

For the preparation of the calibration curve, one of the stock solutions was used and diluted with methanol/water 50/50 v/v + 0.16 mg/ml ascorbic acid to achieve seven calibration solutions 0.50–0.75–4.0–11–38–75-150 mg/L EMB and 1.6–2.5–12.5–38–125–250-500 mg/l RIF.

Quality control (QC) samples are made at three levels (QC Low, medium and high) from a different stock than the calibration solutions at 1.1–11-120 mg/L EMB and 4–40-400 mg/l RIF

All solutions were stored at −40 °C until analysis and are stable for at least 6 months.

**Equipment and settings**

***Supplementary table 10. Pump settings used for ACQUITY UPLC H-Class QSM for rifampicin and ethambutol pharmacokinetic determinations.***

| **Pump settings (****ACQUITY UPLC H-Class QSM; Waters, Milford, MA, USA)** | | | |
| --- | --- | --- | --- |
| **Time (min)** | **Flowrate (ml/min)** | **% A**  10mM ammoniumformate buffer in water pH 4,5 | **% B**  10mM ammoniumformate buffer in methanol pH 4,5 |
| 0 | 0,3 | 98 | 2 |
| 2.05 | 0,3 | 70 | 30 |
| 4.00 | 0,3 | 10 | 90 |
| 5.00 | 0,3 | 10 | 90 |
| 5.10 | 0,3 | 98 | 2 |
| 10.00 | 0,3 | 98 | 2 |
|  | | | |
| Seal wash: 90/10 water/acetonitrile | | | |
| Seal wash period: 5 min | | | |

***Supplementary table 11. Autosampler and column oven settings for rifampicin and ethambutol pharmacokinetic determinations.***

| **Autosampler and column oven (ACQUITY UPLC H-Class SM-FTN; Waters, Milford, MA, USA)** |
| --- |
| Purge solvent: 95/5 water/acetonitrile + 0.1% formic acid |
| Wash solvent: 80/20 water/methanol |
| Injection volume: 1 ul |
| Pre-inject wash time: 5 sec |
| Post-inject wash time: 5 sec |
| Needle placement: 4mm |
| Column Temperature 25 ±1°C |
| Column: Acquity UPLC BEH C18 1.7um 2,1x100mm + assay frit 0.2um 2,1mm |
| Temp. tray: 10°C |

***Supplementary table 12. Ionspray source used for rifampicin and ethambutol pharmacokinetic determinations.***

| **Ionspray source** |
| --- |
| Capillary: 2 kV |
| Polarity positive ion mode |
| Desolvation temperature: 500 °C |
| Desolvation gas flow: 950 L/hr |
| Cone flow: 10 L/hr |

***Supplementary table 13. Mass spectrometer settings used for rifampicin and ethambutol pharmacokinetic determinations***

| **MS settings (XEVO TQS-micro; Waters, Milford, MA, USA)** | | | | | |
| --- | --- | --- | --- | --- | --- |
| **Compound Name** | **Parent (m/z)** | **Daughter (m/z)** | **Dwell (s)** | **Cone (V)** | **Collision (V)** |
| Ethambutol | 205.0 | 116.0 | 0.05 | 26 | 14 |
| Ethambutol ^2^H_4_ | 209.2 | 120.0 | 0.05 | 26 | 14 |
| Rifampicin | 823.2 | 791.4 | 0.05 | 18 | 16 |
| Rifampicin ^2^H_8_ | 831.2 | 799.4 | 0.05 | 18 | 16 |
|  | | | | | |
| **Ionspray source** | | | | | |
| Capillary: 2 kV | | | | | |
| Polarity: positive ion mode | | | | | |
| Desolvation temperature: 500 °C | | | | | |
| Desolvation gas flow: 950 L/hr | | | | | |
| Cone flow: 10 L/hr | | | | | |

**Accessories and disposables**

***Supplementary table 14. accessories and disposable used for rifampicin and ethambutol pharmacokinetic determinations***

| **Name** |
| --- |
| Finnpipette® Air displacement pipette 50-200 µl (ThermoFisher Scientific, Breda, The Netherlands) |
| Finnpipette® Air displacement pipette 5 - 40 µl (ThermoFisher Scientific, Breda, The Netherlands) |
| Finnpipette® Pipette tips 250 µl (ThermoFisher Scientific, Breda, The Netherlands) |
| HandyStep ® Repeater pipette (Brand, Wertheim, Germany) |
| HandyStep ® Repeater pipette tip 5 ml (Brand, Wertheim, Germany) |
| Autosampler vials: TPX micro-vial ND9 TPX clair 0.3ml 32x11.6mm (VWR, Amsterdam, The Netherlands) |
| Autosampler caps: PP Screwcap 9mm pre-slit septum (VWR, Amsterdam, The Netherlands) |
| Safe-Lock tube 1.5 ml (Eppendorf, Nijmegen, The Netherlands) |
| DVX-2500 Multi-Tube Vortexer (VWR, Amsterdam, The Netherlands) |

**Sample preparation**

1. Thaw and mix the (pre) created calibrators and qc’s
2. In safe-lock tubes, dilute the calibrators and qc’s a factor 10 in RPMI 1640+2% FBS (e.g. 40 µl in 360 µl RPMI 1640+2% FBS)
3. Mix the diluted calibrators and qc’s with a multi-tube vortex
4. Pipette in a safe-lock tube: 50 µl sample, diluted QC or calibrator and add 150 µl PP solution
5. Mix with a multi-tube vortex 20 sec. speed 2500 rpm
6. Centrifuge 5 min.(18.620 g)
7. Add to the autosampler vials: 95 µl ascorbic acid 0,9 mg/ml and 75 µl supernatant
8. Close the autosampler vial with a pre-slit cap
9. Mix the autosampler vials with a multi-tube vortex 20 sec. speed 2500 rpm
10. Inject the sample(s) on the chromatographic separation system

**Calculations**

Acquired data was processed using Waters TargetLynx software (version 4.1). The calibration curves were linear plotted as log ratio signal response compound peak area and internal standard peak area versus log concentration.

**Chromatogram**


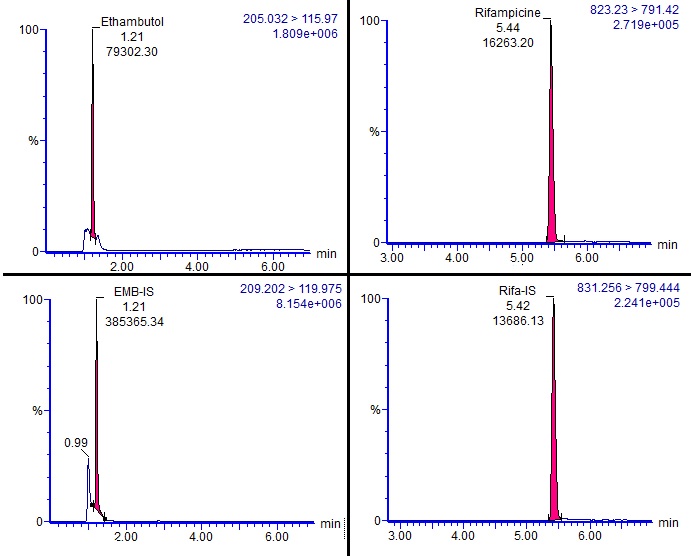


**Supplementary figure 2. Chromatogram of the lowest calibrator with internal standards**

**Validation results**

***Supplementary table 15. Within – and between run accuracy and precision for ethambutol (EMB) and rifamicin (RIF).***

|  | Conc. | Within run (n=5) | | Between run (n=15) | |
| --- | --- | --- | --- | --- | --- |
|  | (mg/l) | Accuracy (%) | Precision (%) | Accuracy (%) | Precision (%) |
| EMB | 0.0480 | 98.0 | 1.22 | 98.8 | 0.94 |
|  | 0.113 | 97.5 | 1.02 | 97.9 | 0.43 |
|  | 1.130 | 97.9 | 1.37 | 98.2 | 1.37 |
|  | 11.30 | 95.1 | 1.25 | 98.9 | 3.42 |
|  | 15.18 | 96.3 | 1.01 | 98.5 | 1.90 |
| RIF | 0.158 | 97.1 | 4.34 | 98.6 | 2.62 |
|  | 0.401 | 95.6 | 3.89 | 98.2 | 2.00 |
|  | 4.006 | 98.5 | 1.63 | 99.5 | 0.97 |
|  | 40.06 | 96.0 | 3.99 | 99.6 | 2.99 |
|  | 49.85 | 96.2 | 2.93 | 97.8 | 1.28 |
